# Does it take three to tango? An unsuspected multimorbidity of CD8^+^ T cell lymphoproliferative disorder, malaria, and EBV infection

**DOI:** 10.1186/s12936-018-2497-9

**Published:** 2018-10-05

**Authors:** Suheyla Ekemen, Ant Uzay, Nuray Bassullu, Emel Dikicioglu-Cetin, Kyoko Matsuda, Umit Ince, Cevayir Coban

**Affiliations:** 1 Acibadem Pathology Laboratory, Istanbul, Turkey; 2 Laboratory of Malaria Immunology, Immunology Frontier Research Center (IFReC), Osaka University, Osaka, Japan; 3 Department of Internal Medicine, Acibadem University Medical Faculty, Istanbul, Turkey; 4 Department of Pathology, Acibadem University Medical Faculty, Istanbul, Turkey

**Keywords:** CD8^+^ T-cell lymphoproliferative disorder, Cutaneous lesions, Hemozoin crystals, Epstein–Barr virus (EBV), *Plasmodium falciparum* malaria

## Abstract

**Background:**

Malaria is known to cause acute and deadly complications. However, malaria can cause unforeseen pathologies due to its chronicity. It increases the risk of endemic Burkitt Lymphoma development by inducing DNA damage in germinal centre (GC) B cells, and leading higher frequency of Epstein–Barr virus (EBV)-infected cells in GCs. EBV is well known for its tropism for B cells. However, less is known about EBV’s interaction with T cells and its association with T cell lymphoma.

**Case presentation:**

A 43-year-old Sudanese male admitted to hospital in Istanbul, Turkey, a non-endemic country, with hyperpigmented painful skin rashes on his whole body. A complete blood count and a peripheral blood smear during admission revealed large granular lymphocytes (LGLs) with abnormally higher CD8 T cell numbers. Additional skin biopsy and pathology results were compatible with CD8^+^ T cell lymphoproliferative disorder with skin involvement. Patient was treated and discharged. However, a pathologist noticed unusual structures in skin tissue samples. Careful evaluation of skin biopsy samples by polarized microscopy revealed birefringent crystalloid structures resembling malarial haemozoin mainly loaded in macrophages and giant histiocytes. After purification of DNA from the skin biopsy samples, nested PCR was performed for the detection of *Plasmodium* parasites and *Plasmodium falciparum* DNA was amplified. Because, the co-presence of EBV infection with malaria is a well-known aetiology of lymphoma, EBV-early RNA (EBER) transcripts were investigated in paraffin-embedded tissue samples and found to be positive in macrophage-like histiocytes.

**Conclusions:**

This is a unique case of malaria and EBV infection in a T-LGL lymphoma patient who presented in a non-endemic country. This case emphasizes the clinical importance of EBV monitoring in T-LGL patients with skin involvement. Notably, *Plasmodium* infection should be examined in patients from malaria endemic regions by pathological and molecular investigations.

## Background

Malaria affects mostly tropical and sub-tropical regions of the world and is known to cause acute and deadly complications such as cerebral malaria, respiratory distress and severe anaemia. However, malaria can cause unforeseen pathologies because of its chronicity [1, 2]. For instance, malaria increases the risk of eBL development by inducing chronic DNA damage in germinal centre (GC) B cells, leading to a higher frequency of Epstein–Barr virus (EBV)-infected cells in GCs [3]. EBV is well known for its tropism for B cells. However, less is known about EBV’s association with T cells, particularly CD8^+^ T cells, in lymphoma. The role of EBV infection as an etiological agent in T cell lymphomas, especially together with CD8^+^ T cell lymphoproliferative disorder, has recently gained attention [4]. Here, a unique case is presented; a multimorbidity of CD8^+^ T cell lymphoma with skin involvement, *Plasmodium falciparum* malaria, and EBV infection, which has not been reported previously.

## Case presentation

A 43-year-old Sudanese male was admitted to Acibadem University Hospital in Istanbul, Turkey with hyperpigmented painful skin rashes on his whole body. He was experiencing these symptoms intermittently for a year and self-medicated himself with non-steroid anti-inflammatory drugs with no fever or other health problems. He had recently experienced joint pains. A complete blood count during admission showed normal erythrocyte counts (5.1 × 10^6^/µL) and Hb levels (13.9 g/dL) with a high white blood cell levels (23.710/µL, of which 85% were lymphocytes) and low neutrophil (10.500/µL) and platelet (128.000/µL) levels. Investigation of a peripheral blood smear revealed 29% large granular lymphocytes (LGLs). Flow cytometric analysis of peripheral blood confirmed that 95% of lymphocytes (CD3^+^/TCRαβ+ population) were positive for pan-T antigens (CD2, CD5, and CD7) and CD8, but negative for CD4 and CD56. Ultrasonography and FDG-PET-CT evaluation of the abdominal area found hepatomegaly, splenomegaly, and hypermetabolic supra-infradiaphragmatic lymph nodes as well as a hypermetabolic spleen. He had a history of malaria, but HCV and HIV tests were negative. These results were compatible with CD8^+^ T cell lymphoproliferative disorder with skin involvement. Therefore, a 0.5-cm-deep skin punch biopsy was performed in an inner part of the leg showing lesions.

LGL leukaemia is a rare lymphoproliferative disease and presents with anaemia, neutropenia, and an increase in the number of LGLs [5]. About 85% of LGL leukaemias are derived from a T cell lineage (T-LGL leukaemic cells express CD3, CD8, CD16, and CD57), while the rest are derived from the natural killer (NK) cell lineage (NK-LGL leukaemic cells express CD2, CD16, CD56, and CD57) [6, 7]. Furthermore, CD8^+^ T cell lymphoproliferative disorder is a very rare form of T-LGL with poorly defined clinical, aetiological, immunophenotypic, molecular and pathological features [6]. Although T-LGL is an indolent disease, it may chronically affect the immune system and cause recurrent infections, symptomatic anaemia, and autoimmune conditions such as rheumatoid arthritis. Prednisone, methotrexate, and cyclosporine have been used for T-LGL treatment. Therefore, the patient with this pre-diagnosis was prescribed methotrexate (20 mg/week) and Prednol^®^ (80 mg) for 6 weeks, and further immunopathological parameters were evaluated in skin lesions.

**Fig. 1:**
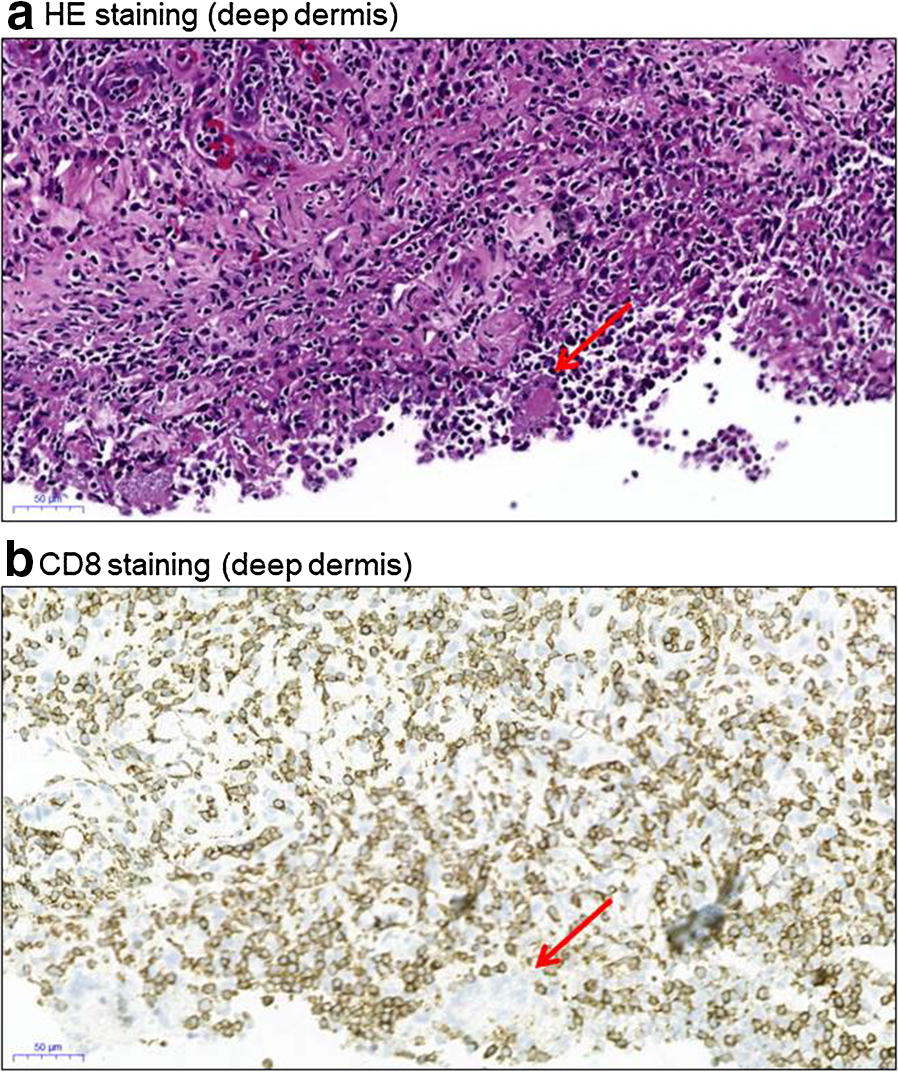
**a** HE staining of a skin biopsy (deep dermis; scale bar: 50 µm). **b** Immunohistochemical staining of CD8 cells in the skin biopsy (deep dermis; scale bar: 50 µm)

**Fig. 2:**
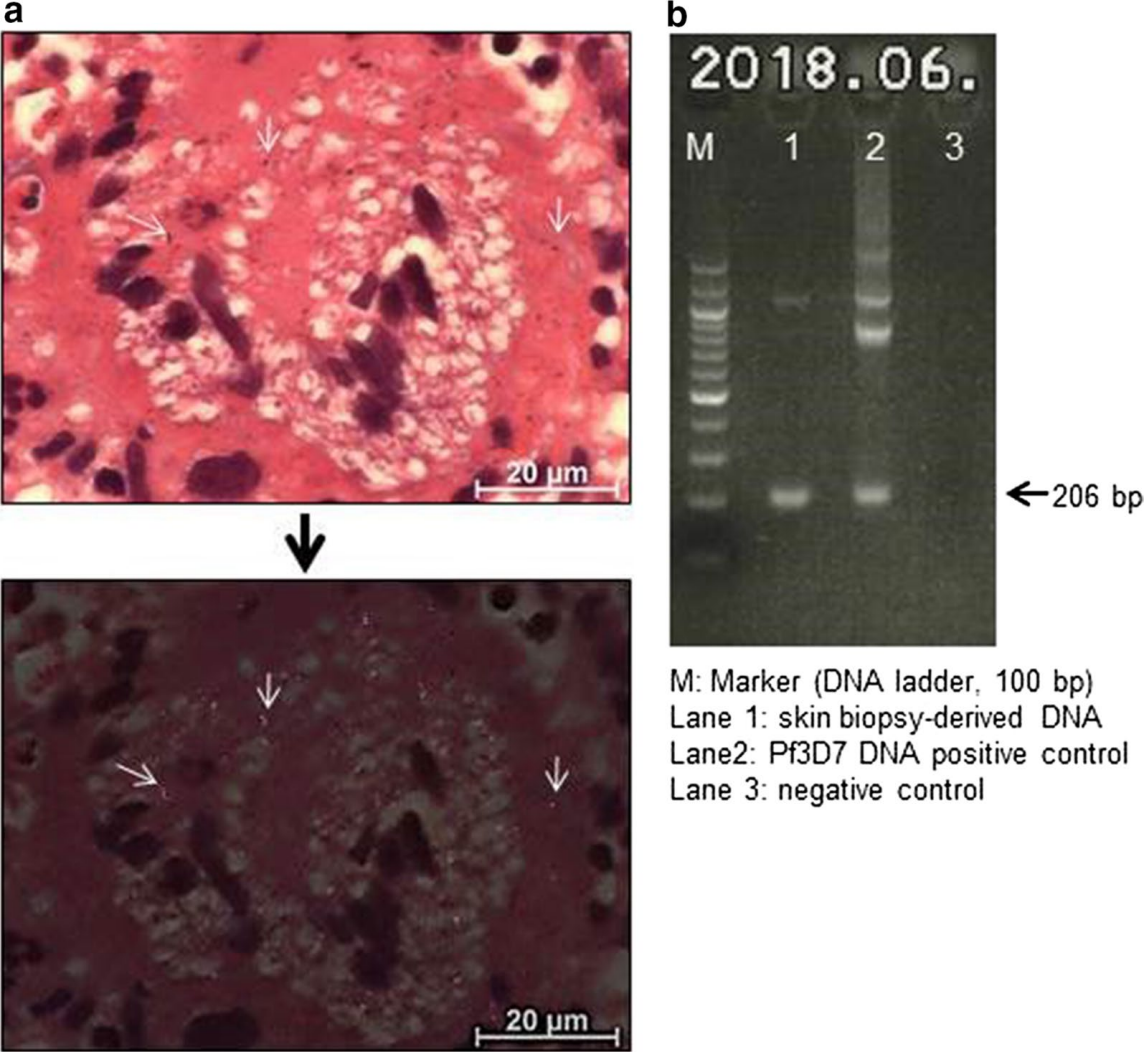
**a** Microscopy of giant histiocytes containing haemozoin crystals with and without polarized light (HE staining; scale bar: 20 µm). **b** Agarose gel electrophoresis of nested PCR products

Microscopic evaluation of skin sections by haematoxylin–eosin (HE) staining showed that the epidermis was minimally spongy and the upper dermis was oedematous with mild perivascular lymphocyte infiltration. However, the deep dermis was infiltrated by intra- and peri-vascular small lymphocytes (Fig. 1a). Standard analysis of paraffin-embedded sections by Benchmark-XT (Ventana Medical Systems) with its inner controls showed that 99% of the total lymphoid population was CD3^+^, CD2^+^, and CD5^+^, among which 90–95% were positive for CD8 (Fig. 1b, red arrow shows a histiocyte with no CD8 staining), while only 5–10% were positive for CD4. Notably, Granzyme B showed a similar staining pattern as CD8 (data not shown). In contrast, CD20 (B cells) and CD56 (NK cells) were negative. At the bottom of the tissue, a few giant multinuclear histiocytic cells were noticed (Fig. 1a, red arrow). Notably, these cells contained small intra-cytoplasmic microorganism-like structures that were not discernibly stained with any dye specific for fungi or bacteria (PAS, Alcian Blue, Grocott’s methenamine silver stain, and Ziehl–Neelsen stain) (Fig. 2a, white arrows). Overall, the patient was finally diagnosed with CD8^+^ T cell lymphoproliferative disorder involving both the periphery and skin.

After this final diagnosis, methotrexate and Prednol were administered for 6 weeks. Six weeks later, blood values had normalized, skin and arthritis symptoms subsided, and the patient was discharged. No other follow-up could be performed because the patient returned to his country. However, because the patient was from Sudan, a malaria endemic region, and the unusual presence of small intra-cytoplasmic microorganism-like structures in histiocytic cells, the pathologist, who had no experience with malaria cases, consulted a malaria specialist at Osaka University, Japan.

Careful evaluation of skin biopsy samples by polarized microscopy revealed birefringent crystalloid structures resembling malarial haemozoin (Fig. 2a, arrows show representative shiny crystalloid structures) [8]. Haemozoin is a by-product of haemoglobin metabolism in *Plasmodium* parasites and readily captured by macrophages and the reticuloendothelial system of the host, which can be easily recognized as birefringent crystals under polarized light [8, 9]. Haemozoin-like structures were mainly loaded in macrophages and giant histiocytes. To further investigate the possibility of asymptomatic submicroscopic chronic malaria infection, we performed nested PCR to detect *Plasmodium* parasites [10]. After purification of DNA from the skin biopsy samples by a tissue DNA extraction kit (NucleoSpin, Macherey–Nagel), *P. falciparum* DNA was amplified (Fig. 2b).

**Fig. 3:**
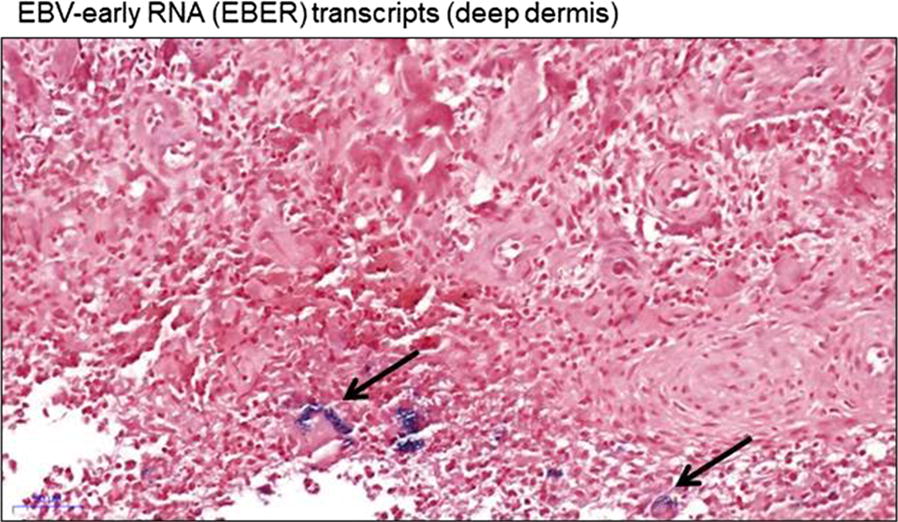
EBER staining of the skin biopsy, (deep dermis; scale bar: 50 µm)

The co-presence of EBV infection with malaria is a well-known aetiology of lymphoma. Hence, EBV-early RNA (EBER) transcripts were investigated in paraffin-embedded tissue samples and found to be positive in macrophage-like histiocytes (Fig. 3).

## Discussion and conclusions

Here, an unexpected and surprising diagnosis of malaria and EBV infection in a T-LGL lymphoma patient who presented in a non-endemic country was reported. Malaria infection has been restricted to the tropical and sub-tropical areas of the world [11], hence, leaving clinicians/pathologists with a lack of knowledge and suspicion about its diagnosis and complications. Although malaria is known to cause acute and deadly complications, however, malaria can cause unforeseen pathologies because of its chronicity [1, 2]. For example, co-infection of *P. falciparum* and EBV is a major risk factor for endemic Burkitt’s lymphoma (eBL) development [3]. In this case report, we additionally evaluated the possibility that the repeated/continuous malaria co-infection with EBV reactivation may also influence T cell maturation and differentiation for an increased risk of cancer [12].

This case has several unique and noteworthy features that complicate the diagnosis and etiology of the multimorbidity. First, the presence of increased LGLs, a higher number and percentage of CD8^+^ T cells in blood, and the presence of skin lesions with similar immunopathological features suggested that this case was not primary cutaneous CD8^+^ T cell lymphoma, but a more general and rare version of T-LGL. Therefore, EBV was not suspected in the aetiology during the diagnosis, although EVB has been investigated in the aetiology of cutaneous T cell lymphomas [13]. Furthermore, the good response to methotrexate and Prednol after 6 weeks of therapy was a successful treatment modality for a patient with a CD8^+^ T cell lymphoma.

Second, previously unseen microorganism-like structures inside the macrophages and giant histiocytes, which could not be stained by any dye, were beyond the expertise of the pathologist for the differential diagnosis of the case. Malaria has been eradicated for more than 50 years ago from many parts of the world including Turkey, resulting in clinicians/pathologists lacking knowledge for its diagnosis and related complications. In the current case, the diagnosis of birefringent haemozoin crystals in the skin tissue led to the suspicion and confirmation of *P. falciparum* infection in the patient by specific nested PCR.

The co-infection of *P. falciparum* and EBV is a major risk factor for eBL development, although the aetiological relationship is somehow enigmatic [3]. One of the important aspects of co-infection in this cancer development is the selective alteration of EBV-specific T cell responses. A recent study [12] has shown that individuals in regions with intense malaria transmission harbor more differentiated EBV-specific CD8^+^ T cell populations that contain less central memory cells, which might allow EBV reactivations. Hence, repeated/continuous malaria co-infection with EBV reactivation may influence T cell maturation and differentiation for an increased risk of cancer. Although it is a speculation, one of these scenarios might be present in the current case.

The third and last unique feature of this case is the presence of EBER in macrophage/macrophage-like giant histiocytes. Although B cells, T cells, and epithelial cells are major targets for EBV, monocytes and macrophages may also play a role in the primary infection or reactivation of EBV [14]. Notably, macrophage/monocytes may express early genes such as EBER, but not latent genes of EBV [14], suggesting that this case had an early EBV infection. The age and origin of patient (Africa, endemic for EBV) may also favour the idea of reactivation of EBV in macrophage/monocytes. However, it is not fully understood what kind of sequential events that might have occurred in this case.

In addition, methotrexate as an immunosuppressive therapy agent is known to activate EBV infection and lymphoproliferative disorders including oral mucosa lesions [15], while removal of methotrexate could reverse the symptoms [16]. However, in this patient, there was no side effect, such as methotrexate-related EBV re-activation. It is possible that methotrexate-related side effects were reversed quickly after stopping therapy due to the limited amount and duration of the therapy. On the other hand, another possibility may arise due to suggested anti-malarial effect of methotrexate [17]. Therefore, there is a possibility that the patient obtained triple benefit from methotrexate therapy for suppressing malaria and CD8-T cell lymphoproliferative disorder and, therefore, reduced EBV re-activation.

In conclusion, this case report emphasizes the clinical importance of EBV monitoring in T-LGL patients with skin involvement. Furthermore, *Plasmodium* infection should be examined in patients from malaria endemic regions by pathological and molecular investigations. EBV co-infection and its possible involvement in T cell lymphoma risk could be another unforeseen complication of chronic malaria infection. Furthermore, the molecular mechanisms underlying the co-presence of *Plasmodium*, EBV infections, and CD8^+^ T cell lymphoma require further investigation in animal models [18].

## References

[1] Coban C, Lee MSJ, Ishii KJ (2018). Tissue-specific immunopathology during malaria infection. Nat Rev Immunol.

[2] Lee MSJ, Coban C (2018). Unforeseen pathologies caused by malaria. Int Immunol.

[3] Thorley-Lawson D, Deitsch KW, Duca KA, Torgbor C (2016). The link between *Plasmodium falciparum* malaria and endemic Burkitt’s Lymphoma-new insight into a 50-year-old enigma. PLoS Pathog.

[4] Ameli F, Ghafourian F, Masir N (2014). Systematic Epstein–Barr virus-positive T-cell lymphoproliferative disease presenting as a persistent fever and cough: a case report. J Med Case Rep.

[5] Greer J. P. (2001). T Cell and NK Cell Lymphoproliferative Disorders. Hematology.

[6] Aribi A, Huh Y, Keating M, O’Brien S, Ferrajoli A, Faderl S (2007). T-cell large granular lymphocytic (T-LGL) leukemia: experience in a single institution over 8 years. Leuk Res.

[7] Cai Q, Chen K, Young KH (2015). Epstein–Barr virus-positive T/NK-cell lymphoproliferative disorders. Exp Mol Med.

[8] Bain BJ (2011). Malaria pigment. Am J Hematol.

[9] Coban C, Yagi M, Ohata K, Igari Y, Tsukui T, Horii T (2010). The malarial metabolite hemozoin and its potential use as a vaccine adjuvant. Allergol Int.

[10] Snounou G, Viriyakosol S, Jarra W, Thaithong S, Brown KN (1993). Identification of the four human malaria parasite species in field samples by the polymerase chain reaction and detection of a high prevalence of mixed infections. Mol Biochem Parasitol.

[11] Murray CJL, Ortblad KF, Guinovart C, Lim SS, Wolock TM, Roberts DA (2014). Global, regional, and national incidence and mortality for HIV, tuberculosis, and malaria during 1990–2013: a systematic analysis for the global burden of disease study 2013. Lancet.

[12] Chattopadhyay PK, Chelimo K, Embury PB, Mulama DH, Sumba PO, Gostick E (2013). Holoendemic malaria exposure is associated with altered Epstein–Barr virus-specific CD8^+^ T-cell differentiation. J Virol.

[13] Novelli M, Merlino C, Ponti R, Bergallo M, Quaglino P, Cambieri I (2009). EpsteinBarr virus in cutaneous T-Cell lymphomas: evaluation of the viral presence and significance in skin and peripheral blood. J Invest Dermatol.

[14] Torii Y, Kawada J, Murata T, Yoshiyama H, Kimura H, Ito Y (2017). Epstein–Barr virus infection-induced inflammasome activation in human monocytes. PLoS ONE.

[15] Kikuchi K, Miyazaki Y, Tanaka A, Shigematu H, Kojima M, Sakashita H (2010). Methotrexate-related Epstein–Barr virus (EBV)-associated lymphoproliferative disorder-so-called “Hodgkin-like lesion”-of the oral cavity in a patient with rheumatoid arthritis. Head Neck Pathol.

[16] Takemori N, Kaneko H, Nakamura M, Kojima M (2012). Complete remission of methotrexate-related Epstein–Barr-virus-associated hodgkin-like lymphoma following withdrawal of MTX coupled with clarithromycin administration. Case Rep Hematol.

[17] Nzila A, Okombo J, Becker RP, Chilengi R, Lang T, Niehues T (2010). Anticancer agents against malaria: time to revisit?. Trends Parasitol.

[18] Matar CG, Jacobs NT, Speck SH, Lamb TJ, Moormann AM (2015). Does EBV alter the pathogenesis of malaria?. Parasite Immunol.

